# Developing Community-Clinical Linkages for Black/African American Older Adults Without Advocates

**DOI:** 10.1093/geroni/igaf122.2987

**Published:** 2025-12-31

**Authors:** Miriam Gofine, Carolyn Berry, Stephanie Zhang, Antoinette Schoenthaler

**Affiliations:** New York University, Brookline, Massachusetts, United States; New York University, New York, New York, United States; New York University, New York, New York, United States; New York University, New York, New York, United States

## Abstract

The terms Older Adults (OAs) Without Advocates (OAWA) or “elder orphans” refer to OAs lacking surrogates in the event of losing decision-making capacity, typically due to absence of kin support. OAWA can face heightened unmet care needs and healthcare access challenges. Prior research identifies limitations in identifying OAWA across clinical and community settings and disparities in healthcare access among Black/African American (B/AA) OAs. While research on B/AA OAWA is limited, sociologists suggest that B/AA OAs receive substantial informal support from networks of church-based community volunteers. Community-clinical linkage models (CCLM) integrate clinical and community settings, including churches, to improve healthcare access for vulnerable populations. While we are unaware of research on age-friendly CCLM, CCLM that meet the specific needs of aging populations, especially those with unmet healthcare needs, such as B/AA OAs, is essential. Guided by a community-engaged framework, this study aimed to identify barriers and facilitators to connecting community-dwelling B/AA OAWA with church-based CCLM through existing church-based informal support networks. We thematically analyzed interviews and focus groups with 18 OAWA and church community volunteers affiliated with predominantly B/AA churches in New York City. Preliminary analyses reveal CISN as highly trusted care network members and facilitators of healthcare access. CISN recognize OAWA as an OA sub-population but do not report unique needs among OAWA in comparison to OA in general. Findings advance ongoing development of an age-friendly CCLM by considering accessibility of CCLM to B/AA OAWA, a uniquely high-need population, and introduce a novel approach to identification of community-dwelling OAWA.

